# Ten-Fold Elevation of Chromogranin A Level Unrelated to a Neuroendocrine Tumor: A Case Report of the Diagnostic Interference of Proton Pump Inhibitors

**DOI:** 10.7759/cureus.46862

**Published:** 2023-10-11

**Authors:** Maha J Alkhayat, Kaamela Davis, Sarah J Atkins, Asad A Sheikh, Muhammad W Saif

**Affiliations:** 1 General Medicine, Alfaisal University College of Medicine, Riyadh, SAU; 2 Medical Oncology/Hematology, Orlando Health Cancer Institute, Orlando, USA

**Keywords:** pancreastatin, neuroendocrine marker, neuroendocrine tumors (net), chromogranin-a, proton-pump inhibitors (ppi)

## Abstract

Chromogranin A (CgA) is a well-known biomarker for neuroendocrine tumors (NETs). However, due to its non-specificity, a proper assessment of CgA test results requires a detailed knowledge of the factors, conditions, and medications influencing its serum concentration. We describe a case of a 61-year-old patient presenting with a mass suspicious of a gastrointestinal NET and an exceedingly high level of serum CgA persistent after mass resection. Following a thorough review of patient's medical history and clinical presentation, along with radiographic and pathological findings, no evidence of a NET was detected. A trial of proton-pump inhibitor (PPI) withdrawal led to a dramatic normalization of CgA level, marking it as the culprit causing this tumor marker elevation. This case highlights the significant impact of PPI use on CgA level, and should incentivize clinicians to provide proper education to patients prior to testing.

## Introduction

Chromogranin A (CgA) is a member of the granin family of proteins and polypeptides. Granins can be found in the secretory granules of endocrine, neuroendocrine, peripheral, and central nervous tissues. CgA plays a major role in the regulation of tissue-specific molecule secretion and enzymatic activity [1]. Moreover, CgA is a precursor of various active substances such as eastatin, catestatin, and vasostatins [1,2].

Due to the extensive presence of CgA in neuroendocrine cells, it is frequently used in clinical practice as a neuroendocrine tumor (NET) biomarker. Its role in the surveillance, treatment monitoring, and disease prognostication remains controversial due to the test's low specificity [3]. Furthermore, CgA's metabolism can be altered by a large number of endogenous and exogenous compounds, including common medications like proton-pump inhibitors (PPIs). Given the high prevalence of PPI use in the United States and the limited data on the influence of PPIs on CgA during the process of NET rule-out, we report a case of a 10-fold increase in CgA level in a patient with gastrointestinal mass and concurrent PPI use.

## Case presentation

A 61-year-old gentleman was referred to the oncology clinic for further evaluation of an abnormal elevation of CgA level. His past history is remarkable for hypertension, dyslipidemia, gastroesophageal reflux disease, and multiple orthopedic procedures for his wrist, shoulder and knee. Medications disclosed at presentation were 5 mg amilodipine, 40 mg atorvastatin, and 20 mg omeprazole. During a routine colonoscopy procedure, he was found to have a mass that was difficult to be biopsied. Consequently, he was admitted to the hospital for an open right hemicolectomy to excise the submucosal mass in the terminal ilium. Upon histopathological testing, the mass showed no evidence of malignancy, but the patient’s recovery course was complicated with ileus and wound dehiscence resulting in the development of a ventral hernia. Patient was scheduled for ventral hernia repair and was found to have an elevated CgA level of 726 ng/mL (reference range <93 ng/mL) during pre-admission testing. Clinical examination at presentation was unremarkable.

As part of the work-up of a suspected NET, a Cu-64 DOTATATE PET/CT showed expected distribution pattern with no localizable pathologic isotope accumulation. Hepatic and renal function tests showed no evidence of abnormalities. Gastroenterological and endocrine biochemical assessment revealed gastrin level of 246 pg/mL (reference range <100 pg/mL), and normal 5-hydroxyindole acetic acid and vasoactive intestinal polypeptide levels. Other tumor biomarkers were undetectable.

**Figure 1:**
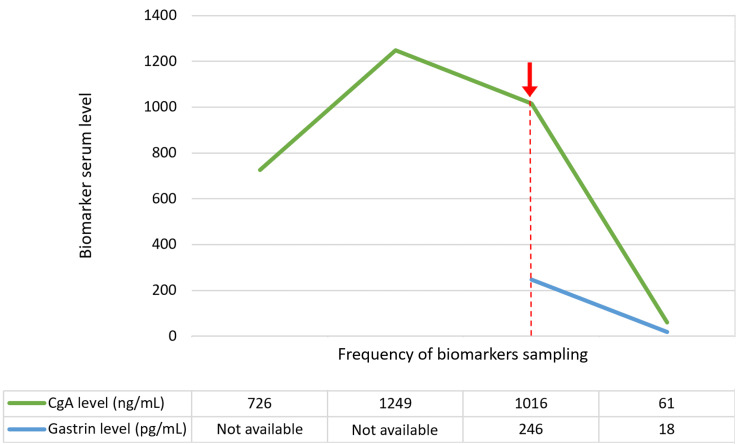
Figure describes elevated serum CgA (green line) and gastrin (blue line) levels, and rapid reduction after two weeks of withdrawal of PPI (marked by arrow and dashed line). CgA: chromogranin A; PPI: proton-pump inhibitor

Repeated CgA levels three to four months apart showed an upward trend measuring 1249 ng/mL and 1016 ng/mL respectively. Following further clinical review and the absence of concerning NET symptoms including vasomotor and B symptoms, the patient was advised to stop omeprazole and repeat biomarkers tests in two weeks. Normalization of CgA and gastrin levels is indicated in Figure 1. He resumed acid-suppressive medication after reassurance of a non-malignant source of elevated CgA.

## Discussion

Our case demonstrated evidence of a dramatic rise and fall of CgA in a patient previously investigated for gastrointestinal NET. Chart review indicated that omeprazole intake was suspected as the cause of the elevation after the initial abnormal result during pre-operative testing, and patient education regarding both benign and malignant causes of elevated CgA was provided. Table 1 lists causes of high CgA levels based on the system involved. Given that initially the patient was being investigated for a submucosal mass in the terminal ilium, NET was still high on the differential diagnoses list, directing the clinicians to conduct a comprehensive pathological, radiographic, and biochemical testing. Upon ruling out of serious causes of high CgA, PPI was withdrawn, causing a large drop in CgA level. Gastrin trends mirroring CgA provide further evidence supporting PPI influence on CgA [4]. 

**Table 1:** Neoplastic and non-neoplastic conditions leading to a positive CgA result based on the system involved. NETs: neuroendocrine tumors; CgA: chromogranin A

System	Neoplastic	Non-neoplastic
Gastrointestinal	NETs: gastrointestinal carcinoid tumors, pancreatic NETs. Non-NETs: colon cancer, hepatocellular carcinoma, pancreatic adenocarcinoma	Chronic atrophic gastritis, chronic hepatitis, liver cirrhosis, pancreatitis, inflammatory bowel disease, irritable bowel syndrome
Endocrine	NETs: pheochromocytoma, pituitary tumors, medullary thyroid carcinoma	Hyperparathyroidism, hyperthyroidism
Cardiovascular	-	Acute coronary syndrome, essential hypertension, cardiac insufficiency/failure, giant cell arteritis
Respiratory	NETs: lung carcinoid tumors, small cell lung cancer, large cell neuroendocrine carcinoma	Smoking-related airway obstruction, chronic bronchitis
Renal	-	Renal insufficiency, renal failure
Reproductive	Non-NETs: breast cancer, ovarian cancer, prostate cancer	Pregnancy, benign prostatic hyperplasia
Nervous	NETs: paraganglioma, neuroblastomas	-
Autoimmune	-	Systemic rheumatoid arthritis, systemic inflammatory response syndrome
Iatrogenic	-	Medications: proton pump inhibitors, histamine-2 receptor antagonists

While a high level of CgA attributed to PPI use is not new in the literature, our case is amongst the very few cases describing extremely high magnitudes of CgA (10X above normal range) due to PPI intake, and rapid drop after discontinuation [5,6].

We draw attention to this case as PPIs are one of the most widely used medications nationwide. It is reported that more than 30% of the United States population use acid-suppressive medications [7]. PPIs are irreversible inhibitors of H+/K+ adenosine triphosphatase enzyme on the surface of gastric parietal cells; they act by reducing gastric acid levels and promoting the release of gastrin. Elevated gastrin level leads to enterochromaffin-like cell hyperplasia, and subsequent increase of CgA levels [8]. In theory, PPIs with a more rapid onset of action (rabeprazole compared to omeprazole) should have a more pronounced effect on gastrin and CgA levels, but there are no studies comparing different PPIs in terms of CgA elevation to support this claim. A similar phenomenon occurs to a lesser degree with histamine type 2 receptor antagonists (H2RAs) [8,9]. A prospective study on 196 patients with known well-differentiated NETs reported that 10% of patients had elevated CgA levels. Amongst these patients, Korse et al. [9] reported an 82% decrease in CgA level after PPI discontinuation, and a 77% decrease after replacing PPI with H2RA. Giusti et al. [10] stated similar values of 81-89% CgA reduction in their study after terminating PPI use.

Providing proper patient education prior to testing reduces the risk of a falsely elevated CgA result. CgA level should be measured in a fasting status, and PPIs should be discontinued for at least two weeks before serum collection [11]. Serial measurement should ideally be conducted using the same assay. Our case used the same time-resolved immunofluorescent assay manufactured by Thermo Scientific (Waltham, MA, USA) for serial CgA measurement. Patients with extremely elevated CgA values, require a longer period (more than two weeks) of PPI discontinuation to reach normal levels [10]. Excessive levels of elevation are seen when patients have a background of hypertension or renal insufficiency, prolonging time to CgA normalization after PPI cessation [10]. An alternative biomarker uninfluenced by PPI is pancreastatin, a peptide precursor of CgA. Pancreastatin assays carry the advantages of being more standardized and strongly predictive of less favorable outcomes in pancreatic and surgically managed small bowel NETs [12].

## Conclusions

There is a lack of united consensus regarding the clinical appropriateness of solely measuring CgA level. Therefore, a judicious interpretation of CgA testing results should be made after addressing pre-testing patient education and preparation, along with their past medical history and clinical findings. Laboratory testing and radiographic modalities can be utilized in conjunction to help exclude causes of a falsely elevated CgA and minimize unnecessary invasive procedures.
